# Efficacy and safety of tyrosine kinase inhibitors plus PD-1 inhibitor in patients with transarterial chemoembolization- refractory hepatocellular carcinoma: a two-center retrospective study

**DOI:** 10.3389/fonc.2023.1231359

**Published:** 2023-11-22

**Authors:** Ya Gao, Haohao Lu, Zhifan Xiong

**Affiliations:** ^1^ Department of Gastroenterology, Liyuan Hospital, Tongji Medical College, Huazhong University of Science and Technology, Wuhan, Hubei, China; ^2^ Department of Radiology, Union Hospital, Tongji Medical College, Huazhong University of Science and Technology, Wuhan, Hubei, China

**Keywords:** hepatocellular carcinoma, TACE-refractory, tyrosine kinase inhibitor, PD-1, camrelizumab, overall survival

## Abstract

**Object:**

To investigate the efficacy and safety of tyrosine kinase inhibitors (TKIs: sorafenib and lenvatinib) plus PD-1 inhibitor (camrelizumab) versus TKIs alone in transarterial chemoembolization-refractory (TACE-refractory) hepatocellular carcinoma (HCC).

**Materials and methods:**

Data of TACE-refractory HCC patients treated with TACE+TKIs+PD-1 inhibitor (TACE+TKIs+PD-1group) (n=57) or TACE+TKIs (TACE+TKIs group) (n=50) from January 2019 to January 2022 were retrospectively collected and analyzed. The differences in overall survival (OS), progression-free survival (PFS), tumor responses (based on modified Response Evaluation Criteria in Solid Tumors) and adverse events (AEs) were compared between the two groups. Potential factors affecting OS and PFS were evaluated by univariate and multivariate analyses.

**Results:**

Compared with the TKIs group, both PFS and OS were prolonged in the TACE+TKIs+PD-1 group (median PFS: 7 months vs. 5 months, *P*=0.007; median OS: 17 months vs. 11 months, *P*=0.002). In multivariate analysis, tumor size and treatment were independent prognostic factors for PFS and OS. The incidence and severity of AEs related to the treatment between the two groups showed no significant difference.

**Conclusion:**

The treatment of TACE combined with TKIs plus camrelizumab demonstrated promising efficacy and safety in TACE-refractory HCC.

## Introduction

Hepatocellular carcinoma (HCC) is a prevalent cancer and ranks third in causing cancer-related deaths globally (1). Despite advancements in HCC surveillance protocols, approximately 80% of patients are diagnosed at intermediate or advanced stages due to the absence of early clinical manifestations, resulting in missed chances for radical resection. Nevertheless, there are multiple treatment options available for HCC, depending on the patient’s clinical situation, tumor stage, and preserved liver function (2).

Transarterial chemoembolization (TACE) is a recommended locoregional therapy for intermediate and advanced HCC (3). TACE may be combined not just with chemotherapy, but also with methods of liver hypertrophy induction (4). However, the efficacy of TACE decreases with the number of procedures, with higher rates of progressive disease observed after each procedure (1, 3, 5, 6). This phenomenon, known as ‘TACE refractory’, was introduced as a new concept by the Japan Society of Hepatology and the Liver Cancer Study Group of Japan in 2014 (7, 8). Since then, it has gained significant attention as a treatment option for advanced TACE-refractory HCC. In order to enhance the effectiveness of TACE, various combinations with liver-directed and systemic therapies such as ablation, radiotherapy, tyrosine kinase inhibitors (TKIs), and immunotherapy have been attempted (9).

Sorafenib and lenvatinib are first-line treatments for advanced HCC, but their survival benefits are limited (10). Immune checkpoint inhibitors (ICIs) targeting programmed death-1 (PD-1) have shown promising results in phase I/II clinical trials such as CheckMate040 and KEYNOTE-224 (11, 12). However, in both phase III trials, CheckMate 459 and KEYNOTE-240, anti-PD-1 therapy did not demonstrate the superiority over the standard of care (13, 14). Recent studies have recommended a combination treatment of anti-PD-(L)1 agent with anti-VEGF antibody or TKIs as the first-line treatment for advanced HCC due to its promising efficacy.

The release of tumor antigens caused by TACE-induced necrosis of tumor tissue may enhance tumor-specific immune responses. Additionally, the hypoxic response after TACE can further stimulate tumor angiogenesis (15). Considering these factors, combining TACE with TKIs and PD-1 inhibitors shows potential for achieving positive outcomes in cases of advanced TACE-refractory HCC (16). The factors mentioned above indicate that a therapy combining TACE with TKIs along with PD-1 inhibitors could yield favorable results in cases of advanced TACE-refractory HCC.

A therapeutic approach that combines TACE, TKIs, and PD-1 inhibitors may yield promising results in the treatment of advanced TACE-refractory HCC. The objective of this study was to retrospectively compare the efficacy and safety of real-world application of TACE+TKIs+PD-1 inhibitor treatment with TACE+TKIs treatment in advanced TACE-refractory HCC.

## Materials and methods

### Patient population

Data on patients in our centers were retrospectively collected from January 2019 to January 2021. The term TACE-refractory refers to the condition where there are two consecutive radiological progressions evaluated by the modified Response Evaluation Criteria in Solid Tumor (mRECIST), even when the chemotherapeutic agents are altered and/or the feeding artery observed on response evaluation CT/MRI is reanalyzed within 1-3 months after effectively conducting selective TACE (17).

The inclusion criteria for the TACE+TKIs and TACE+TKIs+PD-1 groups were as follows: (a) patients aged of 18 years or older; (b) diagnosed with TACE-refractory based on the JSH criteria; (c)Child-Pugh A, B; (d) measurable tumor lesions on computed tomography (CT) or magnetic resonance imaging (MRI); (e) Eastern Cooperative Oncology Group performance score (ECOG PS) of 0 or 1.

The exclusion criteria for this study were as follows: (a) metastatic liver malignant; (b) Child-Pugh class C; (c) any contraindication for therapy with TACE; (d) received with other treatments before progression disease.

According to these criteria, a total of 107 eligible patients with TACE-refractory HCC were included in this study. All patients received TACE and TKIs (including sorafenib and lenvatinib) treatment. Among them, 50 patients who received TACE plus TKIs treatment were assigned to TACE+TKIs group, with an average age of 55.7 ± 11.6 years. Additionally, 57 patients received TACE plus TKIs combined with camrelizumab were assigned to TACE+TKIs+PD-1 group with an average age of 57.5 ± 9.4 years. This study was approved by the ethics committee of Wuhan Union Hospital, Tongji Medical College of Huazhong University of Science and Technology, China.

### TACE procedure

TACE was performed by specialists with at least 10 years of experience in the procedure. To summarize, the patient received local anesthesia using 1% lidocaine, followed by puncturing. Then, the femoral artery was accessed and an arterial sheath was inserted using the Seldinger method. With the assistance of digital subtraction angiography (DSA), a catheter was introduced into the hepatic artery. Additionally, a superselective microcatheter was inserted into the feeding artery of the tumor. Oxaliplatin (100-150mg) and 5-fluorouracil (500-750mg) were infused through the microcatheter. The mixed emulsion contained 10-30ml of hyper-liquefying iodide oil. Furthermore, after hepatic angiography, epirubicin (10-20mg) was injected into the tumor. After diagnosing TACE refractory, all patients received TACE once at least. The TACE procedure would be recommended to repeat once the radiology examination by contrast-enhanced CT within 2 months after previous TACE suggested that lipiodol deposition shrank or residual lesions occurred, which indicated viable lesions or intrahepatic recurrence. TACE treatment is terminated when patient developed trunk portal vein thrombus or severe liver insufficiency (Child–Pugh C). When TACE was discontinued, TKIs + camrelizumab was continuously administrated in TACE+TKIs+PD-1group and TKIs was also continuously administrated in TACE+TKIs group. Symptomatic treatment was administrated in both groups.

### TKIs and camrelizumab administration

Camrelizumab and TKIs were administered within 2 weeks after TACE therapy. Camrelizumab was recommended to be administered intravenously at a dose of 200mg every 3 weeks. Sorafenib was orally administered at a dose of 400mg twice a day. Lenvatinib was recommended to be taken once daily at a dose of 12 mg (≥60 kg) or 8 mg (<60 kg) based on body weight. If patients could not tolerate side effects or had a robust and early response, dose reduction would be determined. In case of serious side effects, drug administration would be interrupted and symptomatic treatment such as glucocorticoids or immune-suppression agents would be administered.

### Follow-up and therapeutic effect evaluation

The clinical characteristics and follow-up data were carefully collected. The first follow-up was conducted at the time of 6 weeks after the first TACE treatment, followed by subsequent follow-ups every 2 or 3 months. Various evaluations were conducted to assess efficacy and safety, including radiology examinations such as contrast-enhanced CT/MRI scans of the abdomen and liver, liver function tests, routine blood tests, and alpha-fetoprotein level assessments. The primary outcome was progress-free survival (PFS), defined as the time interval between the time of TACE refractory and the time of disease progression or death. The secondary outcome was overall survival (OS), indicating the time interval from TACE refractory to the time of death or the last follow-up. The imaging examination results of each patient were evaluated by two diagnostic radiologists with over 10 years of experience. The tumor response was evaluated using mRECIST as follows: Complete response (10) was defined as the disappearance of the enhanced lesion during the arterial phase, reflecting complete tissue necrosis. Partial response (10) was defined as a decrease in the tumor area by at least 30%. Progress Disease (PD) was defined as an increase of at least 20% in the tumor area. And Stable Disease (SD) was defined as neither a sufficient decrease (<30% of tumor area) nor a sufficient increase (<20% of tumor area). The ORR was defined as the sum of CR and PR rate. The disease control rate (DCR) was defined as the sum of CR, PR, and SD rate.

### Safety assessment

Adverse events ware recorded and evaluated based on the Common Terminology Criteria for Adverse Events (CTCAE) version 5.0.

### Statistical analysis

All statistical analyses were performed by SPSS and GraphPad Prism software (SPSS version 24.0 and GraphPad Prism version 8.0). Continuous variables were expressed as medians and ranges, and categorical variables were expressed as frequencies and percentages. The Chi-square test was used to compare categorical variables. The analysis of variance was used to compare continuous variables. The Kaplan-Meier method was used to estimate the survival curve that was compared using the log-rank test. Univariate analyses were performed with the log-rank test. Variables with p<0.1 in univariate analysis were further combined into the multivariate analysis. The multivariate Cox proportional hazard model was used to identify factors independently associated with PFS and OS. P<0.05 (two-tailed) was considered statistically significant.

## Results

### Patient characteristics

From January 2019 to January 2021, 123 patients with TACE-refractory HCC who received TKIs (including sorafenib andlenvatinib) were analyzed. Among them, 107 patients were eligible and enrolled in this study. All patients did not undergo surgical resection/transplantation before therapy. As shown in **Table 1**, The baseline characteristics included age. gender, ALT level, AST level, platelet count, lymphocyte count, neutrophils count, HBV infection, tumor size, tumor number, BCLC stage, portal invasion, extrahepatic expand, cirrhosis, ascites, alpha-fetoprotein (AFP) level, Child-Pugh class, ECOG. In the TACE+TKIs+PD-1 group, 57 patients received TACE plus TKIs combined with camrelizumab treatment. In the TACE+TKIs group, 50 patients received TACE plus TKIs treatment continually until death or the end of follow-up. There were no significant differences in the baseline characteristics between the two groups. The majority of the patients were male (81.3%), and the major etiological factors observed were Hepatitis B virus (HBV) infection (82.2%) and cirrhosis (84.4%).

**Table 1:** The baseline characteristics of patients.

Characteristics	TACE+TKIs+PD-1 (N=57)	TACE+TKIs (N=50)	P value
Age (years)	57.5 ± 9.4	55.7 ± 11.6	0.020
ALT (U/L)	50.4 ± 67.1	39.6 ± 26.1	0.288
AST (U/L)	71.8 ± 62.8	54.6 ± 57.6	0.145
Platelet (*109/L)	172.7 ± 132.7	149.6 ± 80.6	0.288
Lymphocyte (*109/L)	0.8 ± 0.6	1.0 ± 0.7	0.166
Neutrophils (*109/L)	3.6 ± 2.9	3.2 ± 2.1	0.439
Gender			0.504
Male	45	42	
Female	12	8	
HBV infection			0.049
Yes	43	45	
No	14	5	
Tumor size			0.097
≤5 cm	26	15	
>5 cm	31	35	
Tumor number			0.327
1	22	24	
≥2	35	26	
BCLC stage			0.189
B	17	21	
C	40	29	
Portal invasion			0.596
Yes	28	22	
No	29	28	
Extrahepatic expand			0.666
Yes	24	19	
No	33	31	
Cirrhosis			0.119
Yes	45	45	
No	12	5	
Ascites			0.582
Yes	4	6	
No	53	44	
AFP level			0.477
≤200	29	22	
>200	28	28	
Child-Pugh			0.498
A	34	33	
B	23	17	
ECOG			0.570
0	48	40	
1	9	10	
Number of previous TACE			0.948
2-3	30	26	
>3	27	24	
TKIs type			0.805
Sorafenib	31	26	
Lenvatinib	26	24	

ECOG, Eastern Cooperative Oncology Group; BCLC Barcelona Clinic Liver Cancer; HBV, hepatitis B virus; AFP, a-fetoprotein; TACE, transarterial chemoembolization; ALT, alanine aminotransferase; AST, aspartate aminotransferase; TKIs, tyrosine kinase inhibitors.

### Comparison of PFS and OS between the two groups

**Figure 1:**
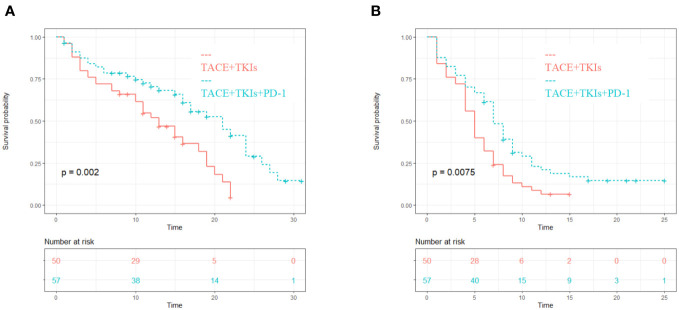
Kaplan-Meier curves of **(A)** PFS and **(B)** OS for patients in the TKIs+C and TKIs groups.

The median PFS of the TACE+TKIs+PD-1 group was 7 months (95% confidence interval [CI] 5.8-8.2), while the median PFS of the TACE+TKIs group was 5 months (95% CI 4.2-5.8). Compared with the TACE+TKIs group, the PFS of TACE+TKIs+PD-1 group was significantly longer(P=0.007). The median OS was 17 months (95% CI 14.2-19.8) in the TACE+TKIs+PD-1 group and 11 months (95% CI 9.9-12.1) in the TACE+TKIs group. Compared with the TACE+TKIs group, the OS of the TACE+TKIs+PD-1 group was significantly longer (P=0.002) (**Figure 1**).

### Prognostic factors affecting PFS and OS

As shown in **Table 2**, the univariate analysis results of factors associated with clinical outcomes suggested that age, ALT level, AST level, Lymphocyte count, Neutrophil count, gender, HBV infection, tumor number, BCLC stage, extrahepatic expand, cirrhosis, ascites, AFP level, and ECOG were not significant predictors for PFS(P>0.10). Factors with P<0.10 including platelet count, tumor size, portal invasion, Child-Pugh class, and treatment were evaluated using the Cox proportional hazards regression model. The multivariate analysis showed that tumor size (≥5cm vs. <5cm) (hazard ratio [HR]=2.106;95% CI:1.322-3.357; P=0.002) and treatment (TACE+TKIs+PD-1 treatment vs. TACE+TKIs treatment) (HR=0.631;95% CI:0.413-0.065; P=0.034) were independently predictor factors of PFS. Moreover, as shown in **Table 3**, univariate analysis indicated that age, ALT level, and AST level, Lymphocyte count, Neutrophil count, gender, HBV infection, tumor number, BCLC stage, extrahepatic expand, cirrhosis, ascites, AFP level, Child-Pugh class, and ECOG were not factors associated with OS(P>0.10). The multivariate analysis indicated that tumor size (≥5cm vs. <5cm) (HR=1.994;95% CI:1.159-3.431; P=0.013) and treatment (TACE+TKIs+PD-1 treatment vs. TACE+TKIs treatment) (HR=0.595;95% CI:0.373-0.950; P=0.030) were independently predictor factors of OS.

**Table 2:** Univariate regression analysis and multivariate regression analysis for PFS.

	Univariate regression analysis		Multivariate regression analysis	
Characteristics	HR (95%CI)	P value	HR (95%CI)	P value
Age (years)	1.001 (0.980,1.022)	0.948		
ALT (U/L)	0.997 (0.990,1.003)	0.279		
AST (U/L)	1.001 (0.998,1.004)	0.640		
Platelet (*109/L)	1.002 (1.000,1.004)	0.022	1.002 (1.000,1.004)	0.050
Lymphocyte (*109/L)	0.981 (0.705,1.366)	0.912		
Neutrophils (*109/L)	1.008 (0.931,1.092)	0.842		
Gender				
Male	Ref			
Female	0.977 (0.576,1.657)	0.931		
HBV infection				
Yes	Ref			
No	0.780 (0.441,1.378)	0.392		
Tumor size				
≤5 cm	Ref		Ref	
>5 cm	2.367 (1.500,3.735)	<0.001	2.106 (1.322,3.357)	0.002
Tumor number				
1	Ref			
≥2	1.376 (0.901,2.101)	0.140		
BCLC stage				
B	Ref			
C	1.389 (0.663,2.911)	0.384		
Portal invasion				
Yes	Ref			
No	0.669 (0.441,1.015)	0.059		
Extrahepatic expand				
Yes	Ref			
No	0.858 (0.566,1.300)	0.470		
Cirrhosis				
Yes	Ref			
No	1.015 (0.573,1.797)	0.960		
Ascites				
Yes	Ref			
No	0.849 (0.427,1.691)	0.642		
AFP level				
≤200	Ref			
>200	0.970 (0.644,1.459)	0.882		
Child-Pugh				
A	Ref			
B	1.515 (0.953,2.408)	0.079		
ECOG				
0	Ref			
1	1.209 (0.722,2.025)	0.471		
Number of previous TACE				
2-3	Ref			
>3	1.025 (0.837, 2.214)	0.510		
Treatment				
TACE+TKIs	Ref		Ref	
TACE+TKIs+PD-1	0.592 (0.390,0.899)	0.014	0.631 (0.413,0.965)	0.034

ECOG, Eastern Cooperative Oncology Group; BCLC Barcelona Clinic Liver Cancer; HBV, hepatitis B virus; AFP, a-fetoprotein; TACE, transarterial chemoembolization; ALT, alanine aminotransferase; AST, aspartate aminotransferase; TKIs, tyrosine kinase inhibitors; PD-1, programmed death-1.

**Table 3:** Univariate regression analysis and multivariate regression analysis for OS.

	Univariate regression analysis		Multivariate regression analysis	
Characteristics	HR (95%CI)	P value	HR (95%CI)	P value
Age (years)	1.006 (0.985,1.029)	0.573		
ALT (U/L)	0.997 (0.990,1.004)	0.380		
AST (U/L)	1.000 (0.996,1.004)	0.964		
Platelet (*109/L)	1.001 (0.999,1.003)	0.195		
Lymphocyte (*109/L)	0.838 (0.583,1.204)	0.339		
Neutrophils (*109/L)	1.011 (0.930,1.098)	0.798		
Gender				
Male	Ref			
Female	0.757 (0.427,1.340)	0.339		
HBV infection				
Yes	Ref			
No	1.061 (0.606,1.858)	0.836		
Tumor size				
≤5 cm	Ref		Ref	
>5 cm	2.434 (1.500,3.948)	<0.001	1.994 (1.159,3.431)	0.013
Tumor number				
1	Ref			
≥2	1.118 (0.723,1.728)	0.616		
BCLC stage				
B	Ref			
C	1.741 (0.781,3.878)	0.175		
Portal invasion				
Yes	Ref		Ref	
No	0.627 (0.404,0.975)	0.038	0.834 (0.517,1.345)	0.457
Extrahepatic expand				
Yes	Ref			
No	0.797 (0.516.1.231)	0.307		
Cirrhosis				
Yes	Ref			
No	1.308 (0.719,2.381)	0.380		
Ascites				
Yes	Ref			
No	0.606 (0.311,1.180)	0.141		
AFP level				
≤200	Ref			
>200	1.174 (0.764,1.805)	0.465		
Child-Pugh				
A	Ref			
B	1.356 (0.839,2.194)	0.214		
ECOG				
0	Ref			
1	1.258 (0.764,2.124)	0.389		
Number of previous TACE				
2-3	Ref			
>3	1.217 (0.641, 2.509)	0.361		
Treatment				
TACE+TKIs	Ref		Ref	
TACE+TKIs+PD-1	0.503 (0.318,0.794)	0.003	0.595 (0.373,0.950)	0.030

ECOG, Eastern Cooperative Oncology Group; BCLC Barcelona Clinic Liver Cancer; HBV, hepatitis B virus; AFP, a-fetoprotein; TACE, transarterial chemoembolization; ALT, alanine aminotransferase; AST, aspartate aminotransferase; TKIs, tyrosine kinase inhibitors; PD-1, programmed death-1.

### Subgroup analysis

**Figure 2:**
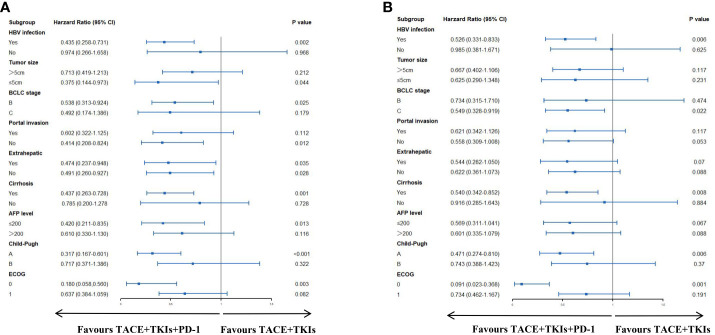
Forest plots of **(A)** progress-free survival and **(B)** overall survival in different patient subgroups. HBV, hepatitis B virus; AFP, alpha-fetoprotein; ECOG, Eastern Cooperative Oncology Group.

As seen in **Figure 2A**, the TACE+TKIs+PD-1 treatment had significantly better PFS than the TACE+TKIs treatment in patients with HBV infection(HR=0.526;95% CI:0.331-0.833;P=0.006), BCLC stage C(HR=0.549;95% CI:0.328-0.919;P=0.022), cirrhosis(HR=0.540;95% CI:0.342-0.852;P=0.008), Child-Pugh class A(HR=0.471,95% CI:0.274-0.810;P=0.006), and ECOG 0(HR=0.091;95% CI:0.023-0.368;P=0.001). As shown in **Figure 2B**, the TACE+TKIs+PD-1 treatment had better OS than the TACE+TKIs treatment in patients with HBV infection(HR=0.435;95% CI:0.258-0.731;P=0.002), tumor size ≤ 5cm(HR=0.375;95% CI:0.144-0.973;P=044), BCLC stage B(HR=0.538;95% CI:0.313-0.924;P=0.025), absence of portal invasion(HR=0.414;95% CI:0.208-0.824;P=0.012), cirrhosis(HR=0.437;95% CI:0.263-0.728;P=0.001), with AFP level ≤200(HR=0.420;95% CI:0.211-0.835;P=0.013), Child-Pugh class A (HR=0.317,95% CI:0.167-0.601;P<0.001), and ECOG 0(HR=0.180,95% CI:0.058-0.560;P=0.003).

### Treatment response

Six patients (10.5%) showed CR, 28 patients (49.1%) showed PR, 17 patients (29.5%) showed SD, and six patients (10.5%) showed PD in the TACE+TKIs+PD-1group. Two patients (4.0%) showed CR, 15 patients (30.0%) showed PR, 12 patients (24.0%) showed SD, and 21 patients (42.0%) showed PD in the TACE+TKIs group. The ORR in the TACE+TKIs+PD-1 group was significantly higher than that in the TACE+TKIs group (59.5 vs.34.0%, *P* = 0.008). The DCR was significantly different between the two treatment groups (89.5 vs. 58.0%, *P*< 0.001).

### Safety assessment

No severe AEs (more than grade 4) or treatment-related deaths were observed. As shown in **Table 4**, the most common AEs were hypertension, elevated TB, fatigue, and hand-foot skin reaction. AEs with grade 3 to 4 occasionally occurred, including hypertension, elevated TB, fatigue, dysphonia, joint pain, and edema. However, these AEs could be effectively managed through dose adjustment. No significantly differences were found in AEs between the two groups.

**Table 4:** Adverse events (>10%) from Any Cause.

Adverse events	TACE+TKIs+PD-1 (N=57)		TACE+TKIs (N=50)	
	All grades N (%)	Grade 3/4 N (%)	All grades N (%)	Grade 3/4 N (%)
Decreased albumin	16/22.8%	0	39/20.5%	0
Hypertension	25/35.7%	10/14.3%	52/27.4%	21/11.1%
Decreased PLT	16/22.8%	0	32/16.8%	0
Bleeding (gingiva)	15/21.4%	0	28/14.7%	0
Elevated TB	22/31.4%	2/2.3%	69/36.3%	13/6.8%
Diarrhea	14/20.0%	0	29/15.3%	0
Fatigue	20/28.6%	4/4.6%	45/23.7%	8/4.2%
Dysphonia	14/20.0%	1/1.4%	23/12.1%	0
Hand-foot skin reaction	22/31.4%	0	89/46.8%	5/2.5%
Elevated creatinine	7/10.0%	0	24/12.6%	0
Prolonged PT	8/11.4%	0	19/10.0%	1/0.5%
Albuminuria/Proteinuria	12/17.1%	0	23/12.1%	0
Decreased appetite	8/11.4%	0	27/14.2%	0
Joint pain	14/20.0%	1/1.4%	28/14.7%	0
Edema	13/18.6%	0	25/13.2%	1/0.5%
Constipation	10/14.3%	0	28/14.7%	0

TB, total bilirubin; PT, prothrombin time; TKIs, tyrosine kinase inhibitors; PD-1, programmed death-1.

## Discussion

In the past decade, the therapeutic reversal of immune exhaustion and immunosuppressive tumor microenvironment (TME) by Immune Checkpoint Inhibitors (ICIs) has been shown effective. Many studies have indicated that ICIs improved the tumor response rates, PFS and OS of patients with advanced HCC (10, 18). In the Check Mate-040 clinical trial, it was demonstrated that patients can experience clinical advantages when administered nivolumab with a dosage of 3 mg/kg (19). Similar to nivolumab, camrelizumab extended the median progression-free survival (PFS) duration up to 9.0 months in advanced hepatocellular carcinoma (HCC) patients undergoing initial TACE treatment (20–22).

Moreover, increasing studies have demonstrated the efficacy of combining TKIs with camrelizumab in treatment of advanced HCC, which may provide a new treatment option for HCC patients (23). For TACE refractory patients, a potential strategy is to combine TACE with PD-1/PD-L1 inhibitors and tyrosine kinase inhibitor (TKI) agents (24, 25). This approach may enhance the immune response against tumors and also inhibit tumor angiogenesis. In this study, our results revealed that patients who received TACE combined with TKIs plus camrelizumab had prolonged PFS and OS compared with the other group who only received TACE combined with TKIs monotherapy (median PFS:7 vs. 5 months; P=0.007; median OS:17 vs. 11 months; P=0.002).

In this study, we found that the treatment of TACE combined with TKIs plus camrelizumab was a protective factor for PFS and OS in TACE-refractory HCC patients. We also observed that tumor size (≥5cm vs. <5cm) was an independent predictor factor for PFS and OS. Other studies have suggested that many factors such as BCLC stage, Child-Pugh class, metastasis, portal invasion are also predictors of PFS and OS (26). However, in our study, although these factors showed an impact on PFS and OS in the univariate analysis, they did not maintain significance in the multivariate analysis after adjusting for other baseline characteristics. This may be attributed to the small sample size, short follow-up time, and inaccurate assessment caused by the retrospective assessment of parameters that are subject to low-reproducibility and high inter-observer variability.

Subgroup analysis suggested that combination of TACE and TKIs with camrelizumab may be more than the treatment of TACE with TKIs for patients with HBV infection, tumor size ≤ 5cm, cirrhosis, Child-Pugh class A, ECOG 0. The AEs in the two groups were mostly mild-to-moderate, and these AEs could be alleviated with supportive symptomatic treatment and dosage adjustment. It demonstrated that this treatment is relatively safe.

Our study has several limitations, including the retrospective design with its inherent drawbacks. Non-availability of key parameters led to exclusion of some patients, which causing the further reduction in sample size. Furthermore, the length of follow-up also had a significant impact on the results. In the future, studies with well-designed, prospective, large-scale randomized and controlled tests are required to confirm our conclusion.

## Conclusion

In summary, the combination of TACE with TKIs plus camrelizumab resulted in extended progression-free survival (PFS) and overall survival (OS) for TACE-refractory HCC patients. The clinical safety of the TACE combined with TKIs plus camrelizumab treatment was demonstrated. All in all, it is efficient and safe for TACE-refractory HCC patients to receive TACE with TKIs plus camrelizumab treatment.

## Data availability statement

The raw data supporting the conclusions of this article will be made available by the authors, without undue reservation.

## Ethics statement

The study was approved by institutional review board of the Union Hospital, Tongji Medical college, Huazhong University of Science and Technology (UHCT-IEC-SOP-016-03-01). The study is being conducted in accordance with standards of Good Clinical Practice and the Declaration of Helsinki. Written informed consent was waived by institutional review board of the Union Hospital, Tongji Medical college, Huazhong University of Science and Technology because of the nature of retrospective study.

## Author contributions

YG, HL, and ZX, and conceived and designed the study. YG contributed significantly to manuscript preparation. All authors contributed to data analysis, drafting or revising the article, have agreed on the journal to which the article will be submitted, gave final approval of the version to be published, and agree to be accountable for all aspects of the work.
